# A Russian Horror

**Published:** 1897-03

**Authors:** 


### A RUSSIAN HORROR.

The Moscow correspondent of *The Lancet* relates an almost incredible instance of the hideous results of ignorance and credulity, which he says is vouched for by an eye-witness who writes to a Southern Russian paper. A peasant woman in the village of Slavyansk has a daughter, aged ten years, who recently suffered from some affliction of the eyes. She consulted a "wise woman" in the village, who gave her the following advice: To procure some gunpowder, put it in the child's eyes and apply a match to it. This advice the mother implicitly followed. The writer of the letter states that he was passing the house at the time, heard the report, and went in to see the cause of it. He found the room full of smoke, and when this had cleared off the wretched child was seen lying in agony, with two cavities where once had been eyes. The mother was also injured by the explosion.—*Medical Record.*

---

Darrier (*Soe. Fran. de Derm. et de Syph.*) states that the affections known as acne cachecticorum, acne scrofulosorum, disseminated folliculitis, bear a close resemblance to each other and form a natural group. Many of these morbid types are frequently observed in the same subject. There is also a constant relation between them and tuberculosis. They are often seen in tubercular individuals and frequently coincident with tuberculosis of the skin. Histological examination has not up to the present brought out this relation, which is so manifest clinically. He proposes for these affections the term *tuberculide.* At the end of his paper he presented two cases to substantiate his views. One was a case of folliculitis in a person suffering from pulmonary tuberculosis, the other of acne cachecticorum with cavities in the lungs.—*Le Progre's Medical.*

